# Inter‐ and intrafractional setup errors and baseline shifts of fiducial markers in patients with liver tumors receiving free‐breathing postoperative radiation analyzed by cone‐beam computed tomography

**DOI:** 10.1120/jacmp.v15i6.4914

**Published:** 2014-11-08

**Authors:** Tao Zhang, Weihu Wang, Yexiong Li, Jing Jin, Shulian Wang, Yongwen Song, Yueping Liu

**Affiliations:** ^1^ Department of Radiation Oncology Cancer Institute (Hospital), Chinese Academy of Medical Sciences, Peking Union Medical College Beijing China

**Keywords:** liver neoplasm, radiotherapy, cone‐beam computed tomography, error, baseline shifts

## Abstract

This study was to evaluate the interfractional and intrafractional setup errors and baseline shifts of golden fiducial markers in patients receiving postoperative radiotherapy (RT) using cone‐beam computed tomography (CBCT) in order to calculate PTV margins for patients with liver cancer. Twelve patients with liver tumors underwent postoperative RT. CBCT images were acquired before and after the treatment. Off‐line vertebral body match and fiducial marker match were used, respectively. The results of vertebral body match represented the setup errors of the patients, while the results of fiducial marker match represented the absolute position errors of the target volume. Baseline shifts of the target volume were calculated as the absolute target position errors minus setup errors. A total of 12 patients with 214 acquisitions of CBCTs were analyzed. Both Σ and σ of setup errors and baseline shifts in left–right (L/R), superior–inferior (S/I), and anterior–posterior(A/P) directions were calculated, including interfractional and intrafractional uncertainties. Planning target volume (PTV) margins were calculated according to margin=2.5Σ+0.7σ. Margins of 1.8 mm, 3.8 mm, and 1.4 mm in L/R, S/I, and A/P directions are needed to compensate intrafractional errors when daily online CBCT correction is used. When CBCT correction with no action level (NAL) protocol is used, PTV margin should be 2.6 mm, 5.9 mm, and 2.6 mm in L/R, S/I, and A/P directions. Margins of 5.5 mm, 14.6 mm, and 7.2 mm were needed to compensate the baseline shifts when electronic portal imaging devices (EPID) or CBCT with bone match is used for online correction of setup error.

PACS number: 87.55.‐x

## I. INTRODUCTION

Hepatocellular carcinoma (HCC) is one of the most common cancers in China, and its incidence is increasing in the world. Among the patients who suffered from HCC, about 54% live in China.^1^, ^2^ Although hepatic resection is the first‐line treatment option, many people are not suitable for these treatments because of the insufficiency of hepatic reserve function or multifocal disease. Technological advances in the planning and delivery of radiotherapy (RT), such as intensity‐modulated radiotherapy (IMRT) or three‐dimensional conformal radiotherapy (3D CRT) combined with image‐guided radiotherapy (IGRT), have enabled dose escalation in RT delivered to patients with unresectable liver tumors, while sparing the adjacent organs and normal liver. Nowadays, radiotherapy plays an important role in the treatment of unresectable or inoperable HCC.^3^, ^4^ Combined with transcatheter arterial chemoembolization (TACE), which is the first choice for patients with unresectable HCC, radiotherapy can improve the survival of the unresectable HCC patients.^5^, ^6^, ^7^ IMRT and 3D CRT can provide conformal dose distribution, while geometrical uncertainties in treatments influence the actually delivered dose distribution. Not only can cone‐beam computed tomography (CBCT), a method of IGRT, correct the setup uncertainties online, but also it can analyze the errors off‐line.^(8)^

Geometrical uncertainties in radiation to the liver tumors include breathing motion, changes in the amplitude of breathing motion, changes in the overall patient position (setup error), changes in the liver tumor position relative to the patient position, and deformation of target volume. Inter‐ and intrafractional shifts in liver position independent of bony anatomy was reported by Guckenberger and his colleagues.^(9)^ These shifts were called baseline shifts, baseline motion or organ motion. Baseline shifts are as important as setup error and breathing motion. Daily online correction using CBCT with tumor matching can correct interfractional setup error and baseline shifts, while planning target volume (PTV) margin is still needed due to the presence of intrafractional errors. But unlike lung cancer, it is difficult to identify the liver tumor in the images of CBCT, which limited the use of CBCT with tumor match. When tumor match is unavailable, CBCT or electronic portal imaging devices (EPID) with bone match is an alternative method. PTV margins compensating the baseline shifts are needed in such a situation because bone match can only correct the setup errors. In some certain circumstances, such as conventional fractionation RT, no action level (NAL) protocol^(10)^ is used instead of daily correction. Perfect NAL protocol can only correct the interfractional systematic error. So the data of random errors and intrafractional errors are needed when calculating the PTV margin for NAL protocol. In some RT institutions of China, correction protocol is not well used and CBCT or EPID is only used for verification at the first fractionation or once per week. PTV margins compensating both baseline shifts and setup errors are needed in such institutions.

Guckenberger et al.^(9)^ used liver contour as a surrogate for the actual target position to analyze baseline shifts. But in our study, fiducial markers placed in the tumor bed were used as a surrogate for actual target position, which is more accurate. The purpose of this study was to evaluate the interfractional and intrafractional setup errors and baseline shifts of golden fiducial markers in liver using CBCT, with which PTV margins using different correction protocols for patients with liver cancer can be calculated.

## II. MATERIALS AND METHODS

### A. Patients

From September 2008 to August 2010, a total of 21 patients with primary liver tumors received postoperative radiation because the tumors were adjacent to big vessels of liver or had close margin in resection. All the 21 patients had 4–6 fiducial markers placed in the tumor bed during the surgery. The fiducial markers were ring‐like and made of silver. They measured 4 mm in diameter. Data from 12 of the 21 patients were available to analyze. Data from the other patients were excluded because of broken data or adjacent fiducial markers to ribs or vertebral bodies. The data of CBCT were backed up and removed from the host computer every month due to huge data and limited storage space. Broken data happened during data restoring for analysis. The bones located in the clipbox influenced the match result and led to incorrect match when using automatic fiducial marker match. Manual match is not used due to the nonrepeatability and interobserver uncertainties. Among the 12 patients, 10 were male. The median age was 50 years old (range: 32–56 years). Of the 12 patients, 8 patients were hepatocellular carcinoma, 3 patients were cholangiocellular carcinoma, and 1 patient was adenosquamous carcinoma.

### B. RT simulation, planning, and treatment

Patients were positioned supine and immobilized in individualized thermoplastic sheet (Klarity, Guangzhou, China) with their arms above their heads. An external marker, Real‐time Position Management (RPM) (Varian Medical Systems, Palo Alto, CA) was used to detect breathing signal. Four‐dimensional CT simulations (Brilliance CT Big Bore; Philips, Healthcare, Andover, MA) were performed during quiet respiration. Neither a breath‐control device nor an abdominal pressure was used. The 4D datasets were sorted out for 10 phase sets within the respiratory cycle. Average CT was generated from the CT images of 10 phases and used as reference CT for planning and matching. Clinical target volume (CTV) included the tumor bed and fiducial markers. Maximum intensity projection (MIP) was used as supplement in order that CTV could include the fiducial markers during all phases of respiration.^(11)^ PTV was expanded by 0.5 cm from the CTV. Simplified IMRTs (sIMRT)^(12)^ were used in all the 12 patients. For sIMRT, the average numbers of segments per beam were no more than five. The segment area was no less than $10\;{\text{cm}}^{2}$. And the monitor units for each segment were no less than 10. Patients received radiation dose of 50–60 Gy/25–30 fractions during five to six weeks.

### C. Acquisition of CBCT images

Patients were treated on linear accelerator (Synergy; Elekta Oncology Systems, Crawley, UK). CBCT was acquired during the first 5 fractions, and 1 fraction per week during the rest weeks to warrant the patients' position. CBCT images were acquired before and after the treatment. For each scan, a full 360° rotation, 120 kV, 1100 mAs, 2 min, approximately 660 projections were obtained in the treatment position. CBCT image sets were compared with the planning reference CT (average CT) using the X‐ray volume imaging (XVI) software (Elekta Oncology Systems). If any translational parameters, left–right (L/R), superior–inferior (S/I), and anterior–posterior (A/P) exceeded 3 mm before treatment, the treatment couch was adjusted and a verification CBCT was acquired to assess residual setup error. A total of 214 CBCT images were acquired and analyzed.

### D. Match methods and errors measurements

**Figure 1:**
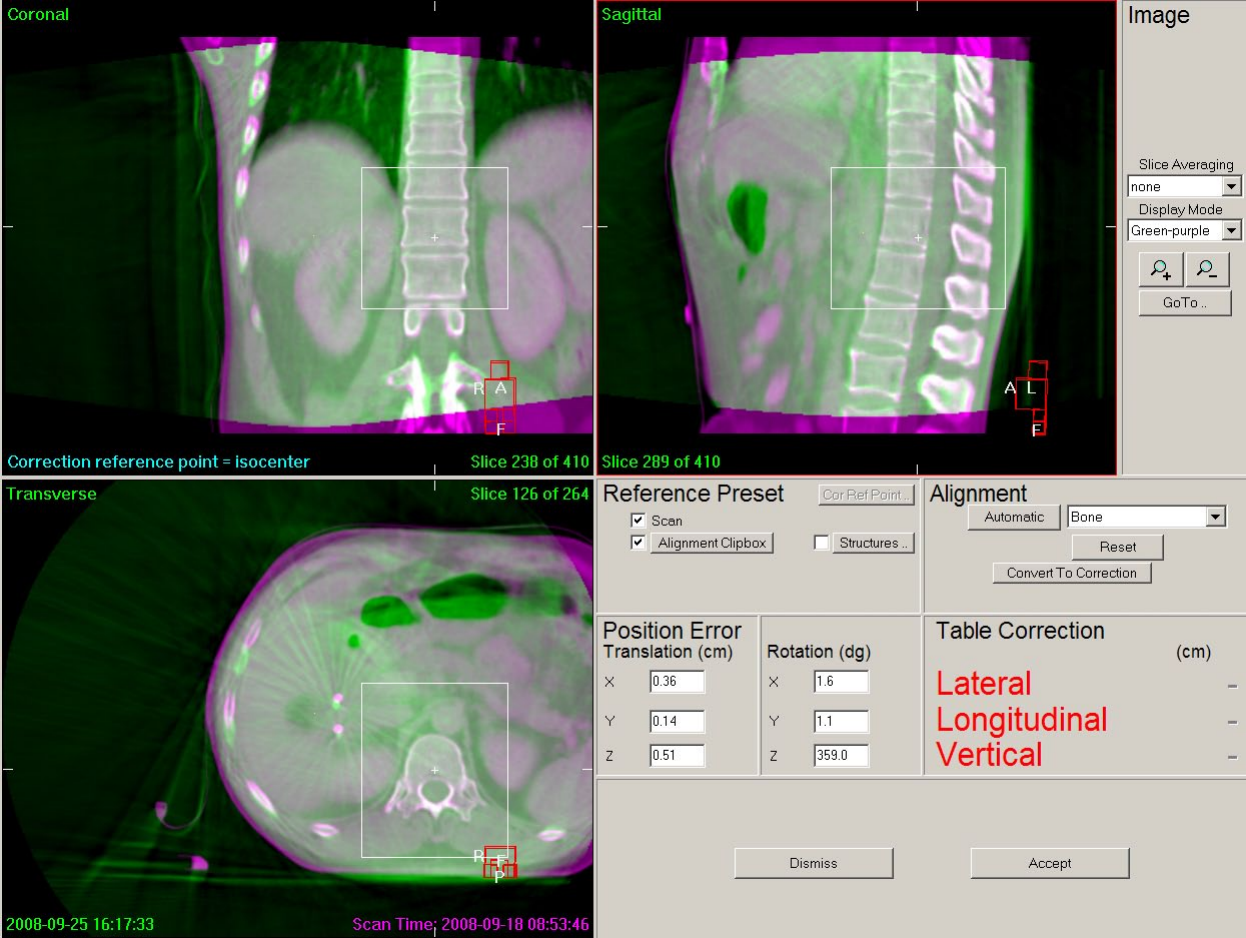
Vertebral body match in which CBCT images are shown in green and reference CT images are shown in purple. The white line is clipbox, which included the vertebral bodies corresponding to the PTV slices. Bones were white when bones from two image sets were completely fit.

**Figure 2:**
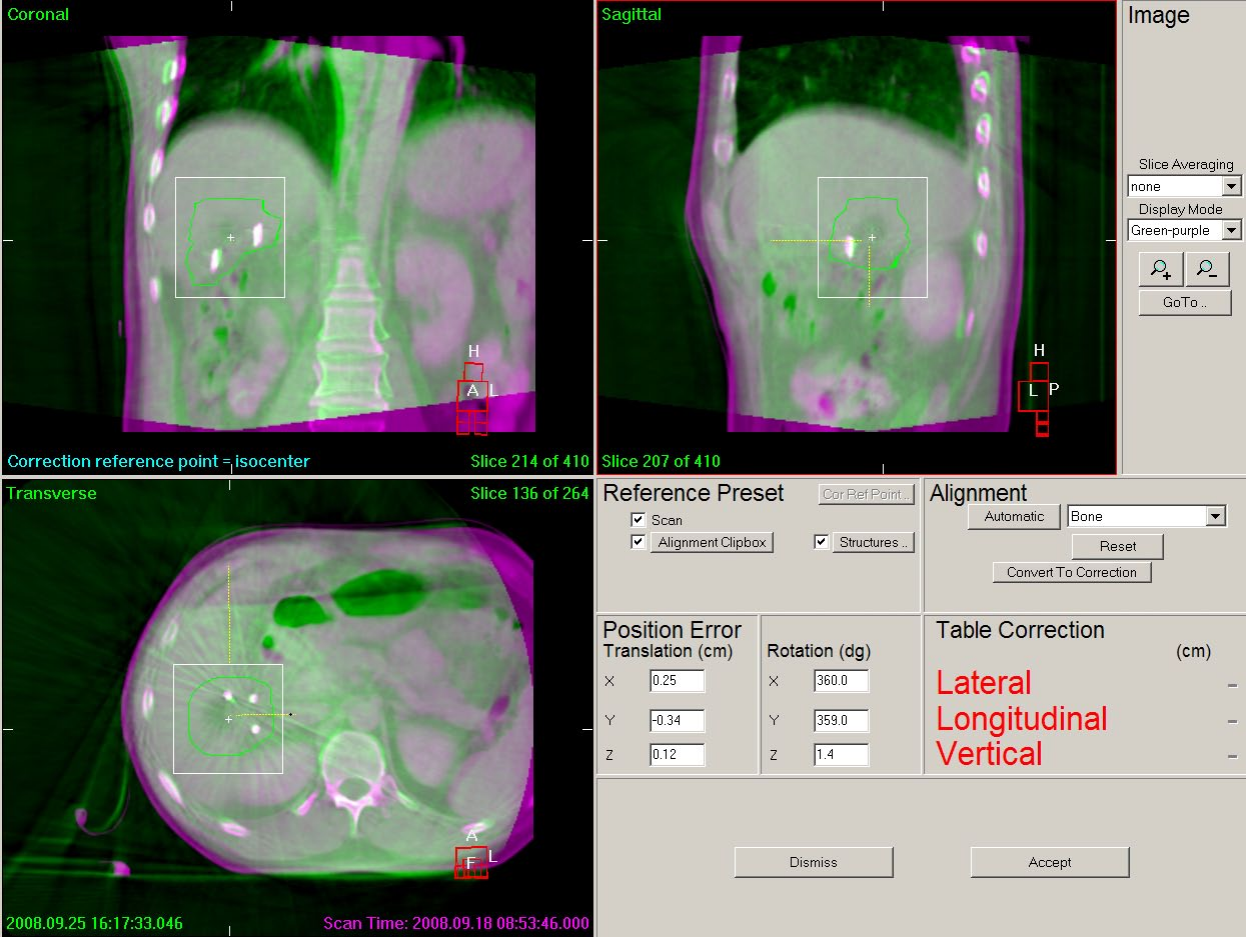
Fiducial marker match in which CBCT images are shown in green and reference CT images are shown in purple. The white line is clipbox, which included the fiducial markers in the tumor bed and excluded the ribs and vertebral bodies. Fiducial markers were white when the markers of two image sets were completely fit.

We retrospectively analyzed the CBCT images with two match methods using different region of interest (clipbox) to calculate the errors. Vertebral body match (automatic bone match) was used and its clipbox included the vertebral bodies corresponding to the PTV slices (Fig. 1). The results represented the setup errors of the patients. Fiducial marker match (automatic bone match) was used and its clipbox included the fiducial markers in the tumor bed and excluded the ribs and vertebral bodies (Fig. 2). The fiducial markers were surrogate of the actual target position and the results represented the absolute position errors of the target volume. The relative target position errors independent of the bony anatomy were calculated as the absolute target position errors minus setup errors, which belonged to the internal errors and named as the baseline shifts of target volume. Both the inter‐ and intrafractional errors were calculated. Intrafractional errors were calculated using the errors acquired from CBCTs before and after same treatment. When treatment couch was adjusted, verification CBCT and CBCT after the treatment is used to calculated intrafractional errors. In our research, most of the rotational errors were less than 2° and the target volumes were spherical‐like, so dosimetric effect of rotational errors was negligible. And only four degrees of freedom (DoF) treatment bed was available, so only translational errors were listed and analyzed in this research.

### E. PTV margin

PTV margins were calculated according to the recipe from van Herk.^13^, ^14^ PTV margin=2.5Σ+0.7σ. The mean (m) and standard deviation (SD) of the inter‐ and intrafractional errors was obtained first for each patient. The group mean error (GME) is the mean of all means of the patients. The SD of the means per patient is an estimator for the SD of systematic error (Σ). The root mean square of all the patients' SD is the mean of the random error (σ). The margin calculated according to this recipe guarantee that 90% of patients in the population receive a minimum cumulative CTV dose of at least 95% of the prescribed dose.

### F. Statistical analysis

SPSS 15.0 was used for descriptive analysis. Microsoft Excel 2007 was used for statistical calculations.

## III. RESULTS

### A. Interfractional errors

Among the 214 CBCTs, 111 CBCTs from 12 patients (7–11 CBCTs per patient) were acquired at the initial setup and used to calculate the interfractional errors. The results of interfractional setup error, absolute target position error, and baseline shifts are listed in Table 1. The margins for compensation of these errors also were calculated and are listed in Table 1.

**Table 1:** Interfractional setup error, absolute target position error, and baseline shifts.

		*GME (mm)*	Σ *(mm)*	σ *(mm)*	*Maximal Error (mm)*	*Margin (mm)*
	L/R	−0.05	1.29	2.22	8.5	4.8
Setup error	S/I	0.60	2.38	3.11	10.6	8.1
	A/P	1.17	2.30	2.09	10.5	7.2
	L/R	−0.04	1.50	1.76	7.4	5.0
Absolute target position error	S/I	−0.35	5.89	4.13	19.0	17.6
	A/P	0.97	1.97	2.42	9.3	6.6
	L/R	0.01	1.36	1.90	7.1	4.7
Baseline shifts	S/I	−0.95	4.69	3.40	14.0	14.1
	A/P	−0.21	2.25	1.48	8.4	6.7

GME = group mean error; Σ = systematic error; σ = random error; L/R = left–right; S/I = :superior–inferior; A/P = anterior–posterior.

### B. Intrafractional errors

Among the 214 CBCTs, 70 pairs of CBCTs (right before and after the treatment) from 10 patients (3–10 pairs per patient) were used to calculate the intrafractional errors.

The results of intrafractional setup error, absolute target position error, and baseline shifts are listed in Table 2, as are the corresponding margins.

**Table 2:** Intrafractional setup error, absolute target position error, and baseline shifts.

		*GME (mm)*	Σ *(mm)*	σ *(mm)*	*Maximal Error (mm)*	*Margin (mm)*
	L/R	−0.18	0.83	1.59	7.0	3.2
Setup error	S/I	0.03	0.64	1.21	5.2	2.4
	A/P	−0.15	0.56	0.90	2.4	2.0
	L/R	0.05	0.46	0.95	3.6	1.8
Absolute target position error	S/I	0.92	1.14	1.38	6.1	3.8
	A/P	−0.42	0.31	0.91	3.1	1.4
	L/R	0.23	0.54	1.78	6.3	2.6
Baseline shifts	S/I	0.89	0.95	1.66	6.0	3.5
	A/P	−0.27	0.69	1.12	3.9	2.5

GME = group mean error; Σ = systematic error; σ = random error; L/R = left–right; S/I = :superior–inferior; A/P = anterior–posterior.

### C. Margin

According to the ICRU report 83^(15)^, the errors should be added in quadrature instead of a linear addition. So the whole errors and margins for both the inter‐ and intrafractional errors were calculated and listed in Table 3.

**Table 3:** The whole margins for setup error, absolute target position error, and baseline shifts.

		Σ *(whole) (mm)*	σ *(whole) (mm)*	*Margin (whole) (mm)*
	L/R	1.53	2.73	5.8
Setup error	S/I	2.46	3.34	8.5
	A/P	2.37	2.28	7.5
	L/R	1.57	2.00	5.3
Absolute target position error	S/I	6.00	4.35	18.0
	A/P	1.99	2.59	6.8
	L/R	1.46	2.60	5.5
Baseline shifts	S/I	4.79	3.78	14.6
	A/P	2.35	1.86	7.2

Σ (whole) = systematic error for both the inter‐ and intrafractional errors; σ (whole) = random error for both the inter‐ and intrafractional errors; Margin (whole) = margin for both the inter‐ and intrafractional errors; L/R = left–right; S/I = superior–inferior; A/P = anterior–posterior.

## IV. DISCUSSION

Geometrical uncertainties include setup error, the movement and deformation of target volume when IMRT or 3D CRT is delivered. Knowing the presence of the errors is not enough. The details of errors are needed to define the volumes of radiotherapy. CBCT is widely used to correct errors online, including setup errors and baseline shifts. In our research, four‐dimensional CT simulation was used. MIP images, average intensity projection (AIP) images (also named average CT images) were reconstructed with the images of 10 phases. Average CT images were used as reference images during image registration in our study according to previous research.^16^, ^17^, ^18^ Average CT images were more like slow CT images, so were free‐breathing cone‐beam CT (CBCT) images, which makes the registration feasible and accurate. The markers and organs from the above two images were comparable. Using the CBCT images, more details about setup errors and baseline shifts can be analyzed.

Setup uncertainties in radiation of thoracic and upper gastrointestinal tumors with different immobilization methods were measured utilizing CBCT by Li and his colleagues.^(19)^ They found that chestboard appeared slightly superior to the evacuated cushion. In patients with upper gastrointestinal tumors immobilized with chestboard, Σ of setup error in the L/R, S/I, and A/P directions were 4.6 mm, 6.3 mm, and 3.0 mm, respectively. And σ of the three directions was 2.5 mm, 6.0 mm, and 1.8 mm. The errors of S/I direction were larger than the other two directions. But our data of the setup errors were a little different. The errors of S/I were not obvious larger than the other two directions, which agreed with the data from Guckenberger and his colleagues.^(9)^ We measured the intrafractional setup errors along with the interfractional setup errors, which showed that the intrafractional setup errors were present.

According to the ICRU report 83,^(15)^ the margin of PTV takes into account both the internal and the setup uncertainties. The setup margin accounts specifically for uncertainties in patient positioning and alignment of the therapeutic beams during the treatment planning and through all treatment sessions. And the internal margin accounts for the uncertainties in size, shape, and position of the CTV within the patient. The errors should be added in quadrature instead of a linear addition, because the margins represent the width of probability distributions.

The internal uncertainties of liver tumors include the changes of the respiratory motion and the changes of the liver tumor position relative to the vertebral bodies. The changes of the respiratory motion should be evaluated by 4D CT or respiratory related CBCT.^(20)^ But the changes of the liver tumor position relative to vertebral bodies, named as baseline shifts or baseline motion, can be analyzed using free‐breath CBCT.

The baseline shifts of the liver,^(9)^ lung,^(21)^ and prostate^22^, ^23^ have been reported. Baseline shifts are as important as the setup uncertainties and respiratory motions, but sometimes are ignored. The baseline shifts of liver or liver tumor may origin from the changes of filling status of lung and gastrointestinal tract, and changes of compression to the abdomen by immobilization device. Data acquired by researchers from Germany^(9)^ used liver as the surrogate for the actual target position in patients with liver tumors receiving SBRT. Respiratory‐related CBCT were used and the exhalation position was preferred for the match. Their data also showed the existence of baseline shifts for both inter‐ and intrafraction. The margin needed for compensation of baseline shifts were 5 mm, 9.5 mm, and 9.3 mm for interfractional changes and 4 mm, 5.6 mm, and 3.9 mm for intrafractional changes in L/R, S/I, A/P directions, respectively.

In our study, we used the fiducial markers as the surrogate for target volume, which is more accurate than the liver. We found that the maximal interfractional baseline shift is 14 mm and intrafractional baseline shift is 6.3 mm. And interfractional baseline shifts in the S/I direction were larger than the other two directions, which maybe means that the target and liver were easily influenced by the movement of diaphragmatic muscle.

With the data of setup errors and baseline shifts, PTV margins using different correcting protocols can be calculated. During the time of CBCT, online tumor match is the optimal choice to eliminate interfractional setup error and baseline shifts. Although liver tumor is difficult to be identified in the images of CBCT, fiducial markers can be implanted in the liver tumor easily. Fiducial markers, as a surrogate for the tumor, can be used in CBCT online correction, which should be a better choice and be more accurate. If daily online tumor match was used, a margin is still needed because of the presence of intrafractional errors, which means 1.8 mm, 3.8 mm, and 1.4 mm is needed in L/R, S/I, and A/P directions. But in patients who should receive conventional fractionation radiotherapy (usually more than 25 fractions), NAL protocol is usually a good choice to correct interfractional systemic errors. So interfractional random errors and intrafractional errors should be taken into account. In this situation, PTV margin should be 2.6 mm, 5.9 mm, and 2.6 mm in L/R, S/I, and A/P directions (data are not listed in the tables).

When the liver tumor is not visible in the CT images and there is no surrogate in the liver, like fiducial markers, tumor match is not feasible. Other surrogates, like vertebral body and diaphragm, will be used during online correction. But more than 70% of the matching results have an absolute error larger than 3 mm.^(24)^ When vertebral body match is used, which can only correct the setup errors, the baseline shifts couldn't be neglected in defining the PTV. The margins needed for compensation of inter‐ and intrafractional baseline shifts were 5.5 mm, 14.6 mm, and 7.2 mm in L/R, S/I, and A/P directions, respectively.

When only EPID is available, digitally reconstructed radiographs (DRR) were used as reference for matching. When fiducial markers are visible in both EPID and DRR, fiducial marker match can be used and it can correct both setup errors and baseline shifts like fiducial marker match of CBCT. So the margins calculated for online tumor match using CBCT is suitable for the fiducial marker match using EPID. If the patient is fiducial marker free, bone anatomy match is used to correct the setup errors, much like the situation of vertebral body match using CBCT. So the margins are needed to compensate for the inter‐ and intrafractional baseline shifts.

PTV margins calculated from the data of absolute target position errors is used in the situation that perfect correction protocol is not available. 5.0 mm, 17.6 mm, and 6.6 mm in L/R, S/I, and A/P directions were needed for interfractional errors, respectively, and 1.8 mm, 3.8 mm, and 1.4 mm were needed for intrafractional errors. The errors should be added in quadrature, so the whole margins in the three directions were 5.3 mm, 18.0 mm, and 6.8 mm, respectively. The margins needed for interfractional error were important. But when margins for both inter‐ and intrafractional errors were calculated, the whole margin increased only 0.2–0.3 mm because the errors were added in quadrature.

## V. CONCLUSIONS

Errors were important and can be analyzed by CBCT and 4D CT. Using CBCT, we acquired the data of absolute errors, setup errors, and baselines shifts in patients with liver tumors. Margins of 1.8 mm, 3.8 mm, and 1.4 mm in L/R, S/I, and A/P directions are needed to compensate for intrafractional errors when daily online CBCT correction is used. When CBCT correction with NAL protocol is used, PTV margin should be 2.6 mm, 5.9 mm, and 2.6 mm in L/R, S/I, and A/P directions. Margins of 5.5 mm, 14.6 mm, and 7.2 mm were needed to compensate for the baseline shifts when EPID or CBCT with bone match is used for online correction of setup error. Margins of 5.3 mm, 18.0 mm, and 6.8 mm were needed to compensate for the setup error and baseline shifts for patients with liver tumors receiving RT without perfect online correction.
